# Exploring the Pathways of Diabetes Foot Complications Treatment and Investigating Experiences From Frontline Health Care Professionals: Protocol for a Mixed Methods Study

**DOI:** 10.2196/54852

**Published:** 2024-04-24

**Authors:** Elisavet Andrikopoulou, Panagiotis Chatzistergos, Nachiappan Chockalingam

**Affiliations:** 1 School of Computing University of Portsmouth Portsmouth United Kingdom; 2 Centre for Biomechanics and Rehabilitation Technologies Staffordshire University Stoke on Trent United Kingdom

**Keywords:** diabetic foot, first-ever diabetic foot ulcer, qualitative research, quantitative evaluation, surveys and questionnaires, telephone interviews, primary care, community care, acute care, education of patients, foot ulcer, exploration, diabetes, foot ulceration, United Kingdom, diabetic foot ulceration, DFU, amputation, complication, complication, perspectives, experiences, health care professionals, barrier, barriers, effective care, foot care, primary ulcers, quality of life

## Abstract

**Background:**

Diabetes affects more than 4.3 million individuals in the United Kingdom, with 19% to 34% developing diabetes-related foot ulceration (DFU) during their lifespan, which can lead to an amputation. In the United Kingdom, every week, approximately 169 people have an amputation due to diabetes. Preventing first-ever ulcers is the most effective strategy to reduce the occurrence of diabetes-related amputations, but research in this space is lacking.

**Objective:**

This protocol seeks to document the experiences and perspectives of frontline health care professionals who work with people who have diabetes and diabetes-related foot problems. Special attention is given to their perceptions of barriers to effective care, their views about barriers to effective and inclusive engagement with people with diabetes, and their experience with the first-ever DFU. Another aspect of the study is the focus on whether clinical management is affected by data sharing, data availability, and interoperability issues.

**Methods:**

This is a mixed methods explanatory protocol, which is sequential, and its purpose is to use the qualitative data to explain the initial quantitative data collected through a survey of frontline health care professionals. Data analysis of quantitative data will be completed first and then synthesized with the qualitative data analysis. Qualitative data will be analyzed using the framework method. This study will use joint displays to integrate the data. Ethical approval has been granted by the ethics committee of Staffordshire University.

**Results:**

The quantitative data collection started in March 2023 and will close in May 2024. The qualitative interviews commenced in November 2023 with volunteer participants who initially completed the survey.

**Conclusions:**

This study’s survey focuses on data interoperability and the interviews focus more on the perspectives and experiences of clinicians and their perceived barriers for the effective management of diabetes foot ulcers. Including a geographically relevant and diverse cohort of health care professionals that spans a wide range of roles and care settings involved in diabetes-related foot care is very important for the successful application of this protocol. Special care is given to advertise and promote participation as widely as possible. The qualitative part of this protocol is also limited to 30-40 interview participants, as it is not realistic to interview higher numbers, due to time and resource constraints.

**International Registered Report Identifier (IRRID):**

DERR1-10.2196/54852

## Introduction

Currently, there are more than 4.3 million people diagnosed with diabetes in the United Kingdom [1], and 19% to 34% of them will develop diabetes-related foot ulceration (DFU) in their lifetime [2]. Out of all DFUs, the literature reports that 10% to 56% will result in amputation [3]. In the United Kingdom, every week, approximately 169 people have an amputation due to diabetes [4,5]. The total direct cost of DFU and diabetes-related foot amputations in England alone is between £837 million (US $1.25 billion) and £962 million (US $1.20 billion) and is steadily rising every year [6]. DFU costs the National Health Service (NHS) more than the 3 most common types of cancer combined [6]; hence, DFU is a major challenge for the financial sustainability of the NHS, and effective prevention could significantly reduce its burden.

Understanding the barriers to effective care and their regional variability is critical for service improvement, leading to fewer diabetes-related foot complications and fewer lower limb amputations [7,8]. A survey of health care professionals working with diabetes-related foot complications, conducted in 2018, highlighted delayed access to specialist care as the most important barrier for effective diabetes-related foot care [9,10]. The key contributors to this problem, which were noted by the responders, included inadequate funding and issues in the areas of referral pathways, patient care, and education [9]. More specifically, about half of the responders indicated a lack of necessary resources. Regarding referrals, the responders of that survey identified inefficiencies in the referral pathways leading to preventable delays. In the area of patient care, the survey indicated a lack of coordination and standardization leading to suboptimal care. The responders also noted that the lack of or ineffective patient education compromises their ability for self-care and leaves them unaware of when to seek help [9].

Structured diabetes education programs are extremely important for promoting effective engagement with health care services and for effective self-management of diabetes-related foot complications [11,12]. To be effective, education needs to be offered to people with diabetes as well as to their families or caregivers [11,12]. It also needs to be designed and delivered in a culturally competent manner [13].

People from ethnic minority groups or socioeconomically disadvantaged communities are disproportionately affected by diabetes and diabetes-related foot complications, experiencing higher morbidity and mortality than majority populations or more affluent communities [13]. Understanding the barriers to effective engagement with these groups is extremely important for effective diabetes-related foot care as well as for robust clinical research.

There is overwhelming evidence that populations from geographical regions that are more actively involved in clinical research tend to benefit from it the most. These benefits include the early adoption and tailoring of new highly effective approaches to care that translate to lower disease prevalence and better patient experience [14,15]. At the same time, regional variations in research participation indicate that the areas and communities with the highest diabetes prevalence are also the least likely to participate in research [16,17]. Ineffective communication and engagement with underserved communities are among the key contributors to this disconcerting discrepancy [14,15], highlighting even further the need to address barriers to effective communication and engagement. Understanding the perceptions of health care professionals about these barriers and how these can be overcome can be helpful to this end.

An exciting new approach for effective DFU care proposed in current literature is to shift medical attention from the management of active DFU to supporting people with diabetes to remain ulcer-free for longer. This can be achieved by focusing efforts to prevent the first-ever DFU from happening [18-20].

Preventing first-ever ulcers (also known as primary ulcers) is the most effective way to reduce the number of diabetes-related amputations and protect the quality of life of people with diabetes. This is because 40% of people with healed first DFU reulcerate within a year (60% reulcerate within 3 years), which significantly increases the risk for amputation [2]. The first-ever DFU incidence is also associated with a 250% increase in the 5-year risk of death [21,22].

Integrated care that combines frequent specialist screening with education and offloading interventions (eg, the use of therapeutic footwear or orthoses) is currently used to prevent recurrent DFU [11,23,24] and is likely to be effective also for the prevention of first-ever ulcers. However, their blanket use across all people with diabetes is practically impossible due to the sheer number of people at risk of their first-ever DFU [18,20,25].

According to current international guidelines, a person with diabetes is considered to be at high risk of DFU when they have had their first-ever DFU or diabetes-related amputation [11,26]. However, as it stands, there is no established method to determine when someone is at high risk of developing their first-ever DFU [11].

Clinical research is needed to develop effective strategies to target preventative care for those at imminent risk for first-ever ulceration [11]. However, studying first-ever ulcers remains significantly more challenging than studying recurrent ulcers. This is due to the scarcity of current and geographically relevant data in the literature to support the design and implementation of research in this area. Moreover, due to their relatively low incidence rate [25], any research on first ulcers is likely to require relatively larger numbers of participants, highlighting the need for effective and inclusive recruitment strategies. To maximize the chances of success, research protocols should be informed by the experience of the health care professionals who will have to lead and deliver this research. This includes identifying strengths to be used and limitations and perceived barriers to participation and recruitment to be addressed [9,27].

In this context, this protocol aims to capture the experience and views of frontline health care professionals working with people with diabetes-related foot complications regarding previously identified barriers to effective care from the literature [9]. These barriers may include the effectiveness of referral pathways, the efficiency of communication between disciplines and NHS services, and the adequacy of patient education The experience of frontline health care professionals regarding the first-ever DFU will be also captured to inform clinical research toward more effective DFU prevention.

The primary objective is to identify the potential areas of improvement regarding DFU care delivery and patient-clinician engagement.

The secondary objectives are to (1) model qualitatively the lived experiences of frontline health care professionals about diabetes foot care and (2) develop a theoretical model that describes the interaction between their lived experience and identified barriers to effective care and engagement with people with diabetes.

## Methods

### Study Design

In agreement with the Equator’s Network mission to improve the reporting of health care research, and in the absence of a comprehensive checklist for mixed methods study design, we have adopted the STROBE (Strengthening the Reporting of Observational Studies in Epidemiology) statement, given that our study is observational [28]. The STROBE statement covers cohort, case-control, and cross-sectional studies and we will follow the relevant statement’s sections about the cohort (for our qualitative data) and cross-sectional (for our quantitative data) studies.

This study uses pragmatism and a mixed methods approach, which has been previously used and supported in the field of health informatics [29,30]. The rationale behind choosing mixed methods is that, based on our pragmatic approach, the best way to achieve the aims and objectives was to mix both quantitative and qualitative data, which complement each other enhancing the results of the study [31,32]. This study uses an explanatory design that is sequential, and its purpose is to use the qualitative data to explain the initial quantitative data.

**Figure 1:**
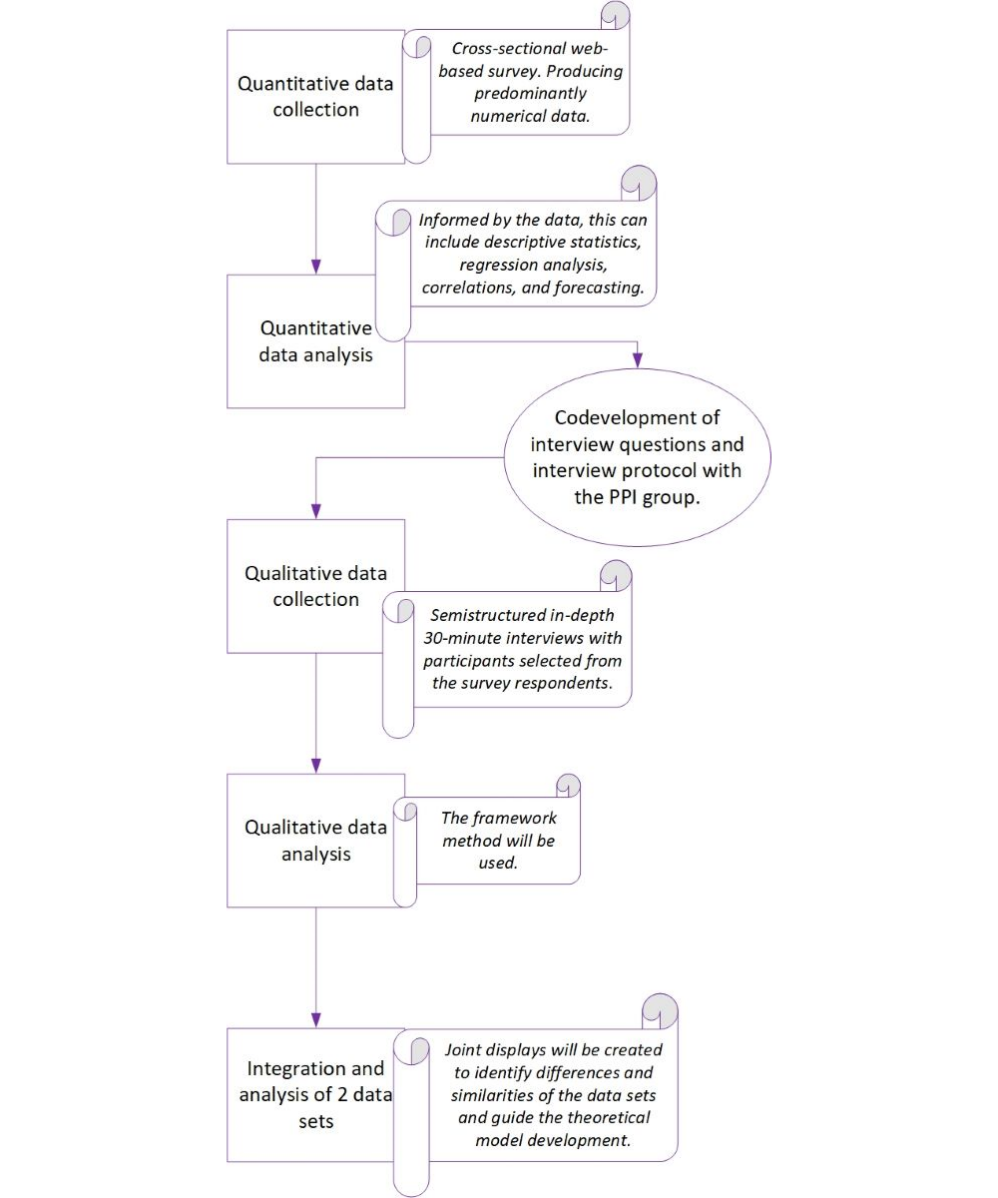
A schematic of the proposed mixed methods sequential explanatory study to capture the experiences and perspectives of frontline health care professionals working in the National Health Service (NHS) in the area of diabetes foot complications. Data collection started in March 2023 and is expected to end in May 2024. PPI: patient and public involvement.

The study has two main components: (1) a survey and (2) semistructured interviews. This study prioritizes the qualitative data as they are used to explain and enhance the findings of the quantitative data [32]. Interview participants were purposefully recruited from the survey responders, mixing the data-gathering methods. Figure 1 illustrates the mixed methods sequential explanatory design stages of this study.

### Patients, Sampling, and Recruitment

Recruitment will occur in 2 stages. The first stage includes the recruitment of survey respondents. We aim to attain between 188 and 270 responses that are required for a maximum assumed error of 6% to 5% (90% Cis), respectively [33].

A recruitment strategy, which encompasses a number of different avenues, has been established to enable us to reach out to a diverse audience and maximize the chances of meeting the required target. This includes the distribution of leaflets in relevant scientific conferences, press releases, social media announcements, and dissemination through a network of collaborating NHS trusts and UK universities (and their alumni associations) that train health care professionals involved in diabetes foot care. Invitation to participate in this survey was also disseminated through the Staffordshire and Shropshire Health Economy Research Partnership [34] through the National Institute for Health and Care Research Clinical Research Network West Midlands [35], special interest groups on diabetes funded by the National Institute for Health and Care Research (Diabesity group), and NHS integrated care boards.

The survey, which is used during this first stage of recruitment, is also used to obtain permission to contact respondents for interviews for the second stage of recruitment. The interviewees will be divided into 3 main cohorts based on their professional work setting (primary, community, or acute care) to discover and model differences in DFU care delivery across contexts. As this study aims for the greatest variation in demographic distribution, the sampling is purposeful [32,36,37]. We intend to interview around 30-40 health care practitioners since the “gold standard” for a purposive sample is to achieve theoretical saturation. It is impossible to predict an absolute number for theoretical saturation; however, based on prior experience and published recommendations, we believe these figures are reasonable given our objectives, scope, and methodology [32,36,38,39].

The interview participants’ selection was based on the following criteria in importance ranking to ensure variation in (1) roles within the care pathway for diabetes foot complications, (2) gender, (3) years of experience, and (4) age group.

Inclusion criteria are (1) participant is willing and able to give informed consent for participation in the study, (2) people aged 18 years or older, and (3) any grade or discipline of health care professional working in diabetes foot care.

Exclusion criteria are (1) inadequate spoken English or unable to participate in a web-based interview, (2) health care professionals who are not working in the diabetes foot care field, and (3) health care professionals not working in NHS.

An assumption has been made that everyone who fills the survey and understands English well. Nonetheless, if we suspect that the interviewee’s English comprehension and articulation are inadequate, we will exclude them and replace this interview.

### Measures

This project will collect quantitative and qualitative data via a web-based survey and further qualitative data from the same group via web-based or telephone interviews.

The survey design was cocreated in conjunction with a patient and public involvement (PPI) group of people with diabetes and diabetes foot complications. The first draft of this survey was cocreated with the 10 health care professionals who also face validated and pilot-tested it. Those health care professionals included a diabetologist, 3 general practitioners, and 6 podiatrists who worked in community, acute, and primary care settings. The survey design can be found in Multimedia Appendix 1. We plan to publish the results of the survey after the analysis is concluded.

Microsoft Excel (Microsoft Corp) will be used for descriptive statistics, while SPSS (IBM Corp) will be used to conduct inferential statistical tests, such as regression analyses and other data-driven tests to assess the association between factors.

For the qualitative component of this protocol, we shall conduct single semistructured interviews (maximum 40 minutes) to explore user experience about the DFU clinical pathway barriers, clinician-patient communication issues, and research participation. We will obtain informed consent and the interviews will be audio recorded and fully transcribed. The interview guide (Multimedia Appendix 2) followed the structure of the NHS Integrated Research Application System guidelines, and it was cocreated with our PPI group.

The framework method [40] will be used for the qualitative data analysis, allowing themes to be produced both inductively (from the interviews) and by adding themes that originate from the survey. The framework method is systematic, comprehensive, and data driven, but it is also adaptable and allows facts to be shown visually [40,41]. The framework method has step-by-step instructions for application, and this study aims to adhere to Gale et al [41] recommendations.

For coding, NVivo (QSR International) will be used. An independent researcher will code a portion of the files (around 25%), and after comparing the themes, the interrater reliability score will be calculated. Following the coding of half of the transcripts, a framework with well-defined and grouped together codes will be created and applied to the remaining transcripts [41]. During data merging, the qualitative data will be interpreted alongside the quantitative.

Having arranged the quantitative and the qualitative data in a format based on thematic relevance to allow merging, further integration will be needed. This study will use joint displays to integrate the data in a similar fashion and to gain a better understanding of the complexity of the observed phenomena [42]. The combined display, should the data collection permit such integration, will have rows representing the participants and columns representing each theme (obstacles in DFU care delivery and research engagement issues, etc), along with the quantitative variables (years of experience, type of employment, etc). This approach may help us better understand the roles of individual themes as enablers or barriers to effective patient care.

### Ethics Approval

Ethical approval for this study has been granted by Staffordshire University’s ethics committee (approval: SU_22_113). All participants provide informed consent before any quantitative or qualitative data are collected.

All potential responders to the survey are first required to read through the participant information sheet and to fill a specific consent form before accessing the main body of the survey. To ensure that no quantitative data are collected without consent, the participants can only access the survey if they agree to all consenting questions (see consent form in Multimedia Appendix 1).

Consenting to take part in the quantitative part of this mixed methods study does not directly qualify a person to participate also in the follow-up interviews. A separate consenting process was followed to this end. More specifically, at the end of the survey, it is explained that based on their responses the research team might want to invite them to take part in a semistructured interview. It is explained that the purpose of this interview will be to ask some more specific questions regarding their response to the survey and that the interview will be on the web or over the phone (see follow-up in Multimedia Appendix 1). The questionnaire responder is then asked to indicate whether they consent to be contacted again to arrange the interview. Answering “no” to this question brings the survey to an end, enabling the responder to submit their answers for analysis. Answering “yes” reveals an additional question where the responders are asked to provide their contact details before submitting their survey responses for analysis and exiting the survey. If they are selected to take part in the qualitative data collection, participants who consent to be contacted again are invited to take part in the semistructured interviews. Consent to be interviewed and to have the interview recorded and transcribed is verbally confirmed at the start of each interview. No qualitative data are recorded for survey responders who do not consent to be contacted again. In addition, no qualitative data are recorded for survey responders who consent to be contacted again but fail to provide explicit consent to be interviewed.

All collected personal information will be kept confidential on a password-protected computer. Only members of the research will have access to this information. The contact details of the people who consent to be contacted again will be used to arrange and conduct the interviews and they will be deleted at the end of data collection. Special care will be given during dissemination to ensure that all data are presented in an anonymized form so that no identification of individual participants is possible.

No compensation or financial incentive will be provided to survey responders. Participants taking part in an interview will receive a £10 (US $12.5) shopping voucher.

### PPI Involvement

People with diabetes and diabetes foot complications have been involved throughout this research to increase transparency, minimize bias, and maximize clinical relevance. All members of the PPI group were recruited based on posters displayed in Stoke-On-Trend community areas and general practitioner practices as well as social media posts. The selection criteria involved people with diabetes and diabetes foot complications, who are consenting adults, and able to express their opinions.

Early in the design of this study, our aim, objectives, and areas of focus of the survey were discussed within a group of 4 people with diabetes and diabetes foot complications. The final questionnaire was designed based on their feedback. The initial core PPI group has since been extended to include 9 people with diabetes and diabetes foot complications. This extended PPI focus group met at Staffordshire University in June 2023 to discuss plans for the semistructured interviews. Discussions in this meeting enabled identifying topics and specific questions that were important to them. These were then included in the interview script. The final interview script was shared with the members of the PPI group during a separate web-based meeting to ensure that their feedback was correctly used.

## Results

So far, this study has had 223 respondents in the web-based survey, and the interview invitations have started. We have invited 30 participants for the interviews, and based on their willingness to be interviewed and their responses, we will keep inviting interviewees, within reason, until theoretical saturation is reached. The study’s data collection will close in May 2024. Initial data analysis has commenced, with results expected to be published by the end of 2024.

## Discussion

The protocol presented here aims to capture the experience and views of frontline health care professionals working with people with diabetes-related foot complications. Emphasis is given to previously identified barriers to effective care regarding the effectiveness of referral pathways, the efficiency of communication between disciplines and NHS services, and the adequacy of available resources and patient education [9]. This protocol also recognizes the need to help people with diabetes remain ulcer-free for longer [18-20]. With this in mind, the experience of frontline health care professionals regarding the first-ever DFU will be also captured to inform clinical research toward more effective DFU prevention.

To this end, a mixed methods explanatory protocol will be implemented. This protocol is sequential, and its purpose is to use qualitative data to explain the initial quantitative data collected through a survey of frontline health care professionals. Data analysis of quantitative data will be completed first and then synthesized with the qualitative data analysis. Compared to relevant literature, this protocol can offer a more comprehensive overview of the perceptions of frontline clinicians regarding diabetic foot care. It is also the first to include the perspective of health care professionals working in primary care [9].

Regarding the limitations of this protocol, even though special care is given to advertise and promote participation as widely as possible, it is likely that dissemination through institutions that are participating in this research will be more effective, leading to greater numbers of responders. These institutions are based in England, and most of them are based in West Midlands. The inability to attain responses with a representative spread across the country could limit the representativeness of results. This study is also limited to the 30-40 interview participants, as it is not realistic to interview higher numbers, due to time and resource constraints.

Once completed, this study can offer new insight into potential improvements regarding DFU care delivery and patient-clinician engagement toward reduced DFU and amputation rates. It will also provide a contextual evidence base for further studies related to DFU care delivery and DFU care workflow.

## Appendix

Multimedia Appendix 1 Survey.

Multimedia Appendix 2 Interview guide.

## Abbreviations

DFU: diabetes-related foot ulceration
NHS: National Health Service
PPI: patient and public involvement
STROBE: Strengthening the Reporting of Observational Studies in Epidemiology

## Data Availability

The data that will be collected as part of this study will be made available upon reasonable request sent to the corresponding author.
