# Supplementary material for: Exploring the Pathways of Diabetes Foot Complications Treatment and Investigating Experiences From Frontline Health Care Professionals: Protocol for a Mixed Methods Study
Source: JMIR Res Protoc. 2024 Apr 24;13:e54852. doi: 10.2196/54852 (PMC11079765; doi:10.2196/54852)
Supplement: Multimedia Appendix 1 [file resprot_v13i1e54852_app1.pdf]

Capturing the experience of frontline clinicians on diabetic foot care

We would like to invite you to participate in this research project. Before you decide whether you want to take part, it is important for you to understand why the research is being done and what your participation will involve. Please take time to read the following information carefully and discuss it with others if you wish. Ask us if there is anything that is not clear or if you would like more information.

The survey will take approximately 10-15 minutes to complete.

* Required

Participant information sheet (1/2)

What is the purpose of the study?

The purpose of this study is to capture the experience and the views of frontline clinicians working with people with diabetes and diabetic foot complications. This information will be used to support robust, effective, and inclusive research and innovation for diabetic foot complications.

Why have I been invited to take part?

You have been identified to participate because you are an active health care professional working in primary care, community podiatry or acute care for diabetic foot complications.

What will happen if I take part?

You will then be asked to complete an online questionnaire. The questionnaire starts with some questions about yourself and your role within the NHS before focusing on your experience with diabetic foot care. Completing the questionnaire will take about 10-15 minutes. The data collected for this study will be anonymised and they will be kept confidential.

Based on your response we might want to ask you some more specific questions to fully understand your perspective and experience. This will be through a semi-structured interview (online/phone). Completing this questionnaire does NOT mean that you automatically agree to be interviewed. At the end of the questionnaire, we will ask you whether (if selected) you are happy to participate in this interview.

Your response to the questionnaire would be greatly appreciated regardless of you agreeing or not to be considered for interview.

Do I have to take part?

Participation is completely voluntary. You should only take part if you want to and choosing not to take part will not disadvantage you in anyway. Once you have read the information sheet, please contact us if you have any questions that will help you decide about taking part or not. If you decide to take part, then the first step before completing the main questionnaire will be to confirm that you consent to taking part.

What if I change my mind about taking part?

At the last page of the questionnaire you will be asked to press “submit” to complete the questionnaire. You are free to withdraw at any point during the process of completing the questionnaire and before pressing “submit”. If you choose to withdraw from the study at that point, then we will not retain any information that you have provided. Withdrawing your data will not be possible once you have submitted your answers.

How is the project being funded?

This study is funded through NIHR CRN West Midlands Strategic Funding

What are the possible risks or benefits of taking part?

There are no risks or intended direct benefits for you taking part in this study.

Participant information sheet (2/2)

Who should I contact for further information?

If you have any questions or require more information about this study, please contact us using the following contact details:

Dr Panos Chatzistergos
Associate Professor of Human Movement Biomechanics
School of Life Sciences and Education
Science Centre
Staffordshire University
Leek Road
Stoke on Trent
ST4 2DF
Email: Panagiotis.chatzistergos@staffs.ac.uk
Telephone: 01782295920

What if something goes wrong?

If this study has harmed you in any way or if you wish to make a complaint about the conduct of the study you can contact the study supervisor or the Chair of the Staffordshire University Ethics Committee for further advice and information:

Dr Tim Horn
Research innovation and impact services
Staffordshire University
Cadman Building, Staffordshire University, College Road
Stoke-on-Trent
ST4 2DE
Email: Tim.horne@staffs.ac.uk
Telephone: 01782 295722

Thank you for reading this information sheet and for considering taking part in this research.

Data handling and confidentiality

Your data will be processed in accordance with the data protection law and will comply with the General Data Protection Regulation 2016 (GDPR). The data collected for this study will be anonymised and they will be kept confidential.

Data Protection Statement

The data controller for this project will be Staffordshire University. The University will process your personal data for the purpose of the research outlined above. The legal basis for processing your personal data for research purposes under the data protection law is a 'task in the public interest' You can provide your consent for the use of your personal data in this study by completing the consent form that has been provided to you.

You have the right to access information held about you. Your right of access can be exercised in accordance with the General Data Protection Regulation. You also have other rights including rights of correction, erasure, objection, and data portability. Questions, comments and requests about your personal data can also be sent to the Staffordshire University Data Protection Officer. If you wish to lodge a complaint with the Information Commissioner's Office, please visit www.ico.org.uk

What will happen to the results of the study?

The data will be used for research purposes and data will be published in either a report or a paper. All personal information we collect during the study will be kept confidential and the data we collect from you will remain anonymous throughout. Information will be kept on a password protected computer, and only the researchers will have access to this information.

Consent form

Please complete this consent form once you have read the information sheet.

1. I have read and understood the information sheet. *

◯ Yes

2. I have been given the opportunity to ask questions, and I have had any questions answered satisfactorily. *

◯ Yes

3. I understand that my participation in this questionnaire is entirely voluntary. *

◯ Yes

4. I consent that data collected could be used for publication in scientific journals or could be presented in scientific forums (conferences, seminars, workshops) or can be used for teaching purposes and understand that all data will be presented anonymously. *

◯ Yes

5. I agree that data may also be audited for quality control purposes. *

◯ Yes

6. I understand that all data will be sorted safely on a password protected computer (electronic data) or locked away securely (hard copies of data) for 10 years before being destroyed. *

◯ Yes

7. I hereby give consent to take part in this survey. *

◯ Yes

Information about you

8. What is your gender *

- ○ Woman
- ○ Man
- ○ Non-binary
- ○ Prefer not to say

9. How old are you (in years)? If you would prefer not to say then please type "N/A". *

[ ]

10. How many years of experience do you have working within NHS? *

- ◯ up to 5 years
- ◯ 6 years to 10 years
- ◯ 11 years to 15 years
- ◯ 16 years to 20 years
- ◯ 21 years to 25 years
- ◯ more than 25 years
- ◯ Prefer not to say

11. Choose one option that best describes your ethnic group or background *

- ◯ White - English/Welsh/Scottish/Northern Irish/British
- ◯ White - Irish
- ◯ White - Gypsy or Irish Traveller
- ◯ Any other White background
- ◯ Mixed/Multiple ethnic groups - White and Black Caribbean
- ◯ Mixed/Multiple ethnic groups - White and Black African
- ◯ Mixed/Multiple ethnic groups - White and Asian
- ◯ Any other Mixed/Multiple ethnic background
- ◯ Asian/Asian British - Indian
- ◯ Asian/Asian British - Pakistani
- ◯ Asian/Asian British - Bangladeshi
- ◯ Asian/Asian British - Chinese
- ◯ Any other Asian background
- ◯ Black/ African/Caribbean/Black British - African
- ◯ Black/ African/Caribbean/Black British - Caribbean
- ◯ Any other Black/African/Caribbean background, please describe
- ◯ Prefer not to say

12. Where are you based? *

- ◯ Scotland
- ◯ Wales
- ◯ Northern Ireland
- ◯ England, North East
- ◯ England, North West
- ◯ England, Yorkshire and The Humber
- ◯ England, East Midlands
- ◯ England, West Midlands
- ◯ England, East of England
- ◯ London
- ◯ England, South East
- ◯ England, South West
- ◯ Prefer not to say

Your role within the care pathway for diabetic foot complications

13. Please select the option that describes best your role within the care pathway for diabetic foot complications. *

- ◯ Primary care - GP
- ◯ Primary care - Nurse
- ◯ Primary care - Allied health practitioner
- ◯ Community care - Podiatrist
- ◯ Community care - Podiatry assistant
- ◯ Acute care - Diabetologist
- ◯ Acute care - Vascular surgeon
- ◯ Acute care - Microbiologist
- ◯ Acute care - Orthopaedic surgeon
- ◯ Acute care - Podiatrist
- ◯ Acute care - Orthotist
- ◯ Acute care - Nurse
- ◯ Other

14. If you work in acute care, then are you part of a multidisciplinary foot care service? *

- ◯ Yes
- ◯ No

15. If you answered YES in the previous question then please select from the list below the clinical specialties that are present in your multidisciplinary foot care team. *

- ☐ I am not a member of a multidisciplinary foot care team
- ☐ Diabetes specialist nurse
- ☐ Diabetologist
- ☐ Microbiologist
- ☐ Orthopaedic surgeon
- ☐ Podiatrist
- ☐ Tissue viability nurse
- ☐ Vascular surgeon
- ☐ Other

Environment

16. How do people with diabetic foot complications get access to your service? Please select as many options from the list below that are relevant to your practice. *

- [ ] They are referred to my service by their GP
- [ ] They are referred to my service by community podiatry
- [ ] They are referred to my service by other services within acute care
- [ ] People present to my service with a health concern
- [ ] Through community-based screening organised by 3rd sector organisations (diabetes charities, care homes etc.)
- [ ] Other

17. How easy is it for you to adhere to NICE guidelines relevant to your service? *

- ○ Extremely easy
- ○ Somewhat easy
- ○ Neutral
- ○ Somewhat not easy
- ○ Extremely not easy
- ○ I don't know what the NICE guidelines are for my service

18. How easy is it to refer patients to other services related to diabetic foot care? *

- ◯ Extremely easy
- ◯ Somewhat easy
- ◯ Neutral
- ◯ Somewhat not easy
- ◯ Extremely not easy
- ◯ I do not refer people to other services as part of my role

19. Are you aware of any equality and diversity issues with regards to NHS staff? *

- ◯ Yes
- ◯ No
- ◯ Prefer not to say

20. If you answered YES in the previous question, then please indicate the characteristics that might lead to a member of the NHS being disadvantaged. You can select as many answers as you want. You need to select at least one answer to proceed. If you would prefer not to answer this question then please select "prefer not to say". *

- [ ] Race
- [ ] Sexual orientation
- [ ] Sex
- [ ] Other
- [ ] Age
- [ ] Marriage and civil partnership
- [ ] Mental health
- [ ] Pregnancy and maternity
- [ ] Religion or belief
- [ ] Gender reassignment
- [ ] Prefer not to say
- [ ] First language
- [ ] Disability
- [ ] Socioeconomic status

Resources

21. Do you have the equipment, the technology and the infrastructure you believe is needed to offer effective diabetic foot care? *

- ◯ Yes
- ◯ No
- ◯ Prefer not to say

22. Does your service have the personnel that is needed to offer effective diabetic foot care? *

- ◯ Yes
- ◯ No
- ◯ Prefer not to say

Primary diabetic foot ulcers

23. How often do you see people with their first diabetic foot ulcer or with a suspected first ulcer within your service? *

- ○ Daily
- ○ Weekly
- ○ Monthly
- ○ Seasonal
- ○ Yearly
- ○ Never

24. How do people with their first ulcer (or a suspected first ulcer) come to your service? Please select as many options from the list below that are relevant to your practice. *

- ☐ I do not see people with first ulcers in my service
- ☐ They come on their own directly to my service
- ☐ They are referred to my service by primary care
- ☐ They are referred to my service by services within acute care
- ☐ Through community-based screening
- ☐ Other

Data

25. Does your service record data on diabetic foot complications using a structured data collection form? For example a form with specific questions to answer and/or fields to fill in. *

- ◯ Yes
- ◯ No
- ◯ I do not know/ prefer not to say

26. Does your service contribute data to a patient database? Please select as many options from the list below that are relevant to your practice. *

- ☐ National diabetic foot care audit (NDFA)
- ☐ Clinical Practice Research Datalink (CPRD)
- ☐ Other national database
- ☐ Other regional database
- ☐ Data are stored in a database for the entire NHS Trust for which I work
- ☐ Data are stored in a database that is specific to my service
- ☐ I do not know/ prefer not to say
- ☐ Other

27. Consider the hypothetical scenario where you want to find out when a person with history of diabetic foot ulceration developed their first ever ulcer. How easy or difficult do you think it would be to look back into the patient's data and find the date of their first diabetic foot ulcer? *

- ◯ Extremely easy
- ◯ Somewhat easy
- ◯ Neutral
- ◯ Somewhat not easy
- ◯ Extremely not easy

28. Based on your experience is there sharing of data between primary care, community podiatry and acute care on diabetic foot complications? *

- ◯ Yes
- ◯ No
- ◯ I do not know

29. If there is shared data what can we do better? If you prefer not to say then please type "N/A". *

30. What additional patient data would you like to have readily available to you in your service in terms of diagnosing and treating the diabetic foot? If you already have access to all the data that you need, then please type "none". If you prefer not to say then please type "N/A". *

31. How easy is it for you to access patient data from other NHS services? *

- Extremely easy
- Somewhat easy
- Neutral
- Somewhat not easy
- Extremely not easy

32. Based on your personal experience, how important is data sharing between NHS services? *

- Extremely important
- Somewhat important
- Neutral
- Somewhat not important
- Extremely not important

33. Based on your experience how easy is data sharing within the NHS? *

- ◯ Extremely easy
- ◯ Somewhat easy
- ◯ Neutral
- ◯ Somewhat not easy
- ◯ Extremely not easy

Patients

34. Are you aware of any equality and diversity issues with regards to diabetic foot patients? *

- ◯ Yes
- ◯ No
- ◯ Prefer not to say

35. If you answered YES in the previous question, then please indicate the characteristics that might lead to a patient being disadvantaged. You can select as many answers as you want. You need to select at least one answer to proceed. If you would prefer not to answer this question then please select "prefer not to say". *

- [ ] Other
- [ ] Socioeconomic status
- [ ] English language level
- [ ] Gender reassignment
- [ ] Religion or belief
- [ ] Sex
- [ ] Prefer not to say
- [ ] Age
- [ ] Marriage and civil partnership
- [ ] Sexual orientation
- [ ] Mental health
- [ ] Disability
- [ ] Race
- [ ] Pregnancy and maternity

36. Based on your experience, are patients aware of the standards of care they should be receiving for diabetic foot complications? *

- ◯ All of them are aware
- ◯ Most of them are aware
- ◯ Most of them are NOT aware
- ◯ None of them are aware
- ◯ I do not know/ prefer not to say

37. Based on your experience are some groups of people or communities better informed than others about the standards of care they should be receiving? *

- ◯ Yes
- ◯ No
- ◯ I do not know/ prefer not to say

38. If you answered YES in the previous question, then please briefly indicate (according to your experience) which groups of people or communities are NOT as well informed as others about the standards of care they should be receiving. If you do not want to answer then please type "N/A". *

39. Based on your experience do patients receive the necessary education on self-care for diabetic foot complications? *

- ◯ All of them do
- ◯ Most of them do
- ◯ Most of them don't
- ◯ None of them do
- ◯ I do not know/ prefer not to say

40. Based on your experience are some groups of people or communities better informed than others on self-care for diabetic foot complications? *

- ◯ Yes
- ◯ No
- ◯ I do not know/ prefer not to say

41. If you answered YES in the previous question, then please briefly indicate (according to your experience) which groups of people or communities are NOT as well informed as others on self-care for diabetic foot complications? If you do not want to answer then please type "N/A". *

42. What are the most important barriers hindering disadvantaged groups of people or communities engage effectively with the NHS? If you do not want to answer then please type "N/A". *

Follow-up

Based on your response we might want to ask you some more specific questions to fully understand your perspective and experience. This will be through a semi-structured interview (online/ phone). In this section, we are asking you to tell us whether you would be interested to take part in this follow-up process.

43. If I am selected for the follow-up interviews, then I would be interested to take part? *

○ Yes

○ No

44. If you selected YES in the previous question, then please provide a telephone number and/or email address we can use to contact you. *

Please submit your answers.

You will not be able to withdraw your data from the study once you press “submit”.

This content is neither created nor endorsed by Microsoft. The data you submit will be sent to the form owner.

Microsoft Forms
