# Supplementary material for: Exploring the Pathways of Diabetes Foot Complications Treatment and Investigating Experiences From Frontline Health Care Professionals: Protocol for a Mixed Methods Study
Source: JMIR Res Protoc. 2024 Apr 24;13:e54852. doi: 10.2196/54852 (PMC11079765; doi:10.2196/54852)
Supplement: Multimedia Appendix 2 [file resprot_v13i1e54852_app2.pdf]

Elisavet Andrikopoulou, Panagiotis Chatzistergos, Nachiappan Chockalingam, **A mixed methods case study protocol to identify the experiences of frontline healthcare professionals working with people with diabetic foot complications.**

## Patient Interview Guide

Thank you for your time today. My name is _____, I am a ____ at ____ and I will be conducting your interview today. May I first check that you are still willing to do this interview and for me to record it? The interview should take no more than 25 minutes, is that ok?

[If participant declines, thank and close the conversation. If they are willing to re-schedule, try to find a suitable time within the next week or so.]

As a reminder we would like to inform you that the aim of this study is to explore the experience of frontline NHS clinicians regarding diabetic foot ulcer care. One of the reasons for doing this research is to understand why there is an apparent discrepancy between the regions that have high prevalence of diabetic foot complications and the regions that participate in research in the UK. We also want to explore whether focusing on the prevention of first ever ulcers is feasible.

## 1. Initial Questions

Could you please verify your job title?

How often do you see patients with diabetic foot ulcers in your practice?
> [Pause, listen, probe if answer is vague. i.e. what do you mean every so often?]

Are these usually first-ever ulcers? Do you see any people with first-ever ulcers?
> [Pause, listen, probe: How often?]

Do you usually treat them within your service or refer them to another service?
> [Pause, listen, probe: Where do you often refer them to?]

In your opinion do your diabetes foot ulcer patients know that they have an ulcer when they come to see you?
> [Pause, listen, probe: why do you say that?]

Do you have experience from first-ever ulcers and from recurrent ulcers?
> [Pause, listen, if **YES** probe: is there any difference between these two regarding the patient journey through the system?]
> [Pause, listen, probe: Is there a difference between the people presenting with a first-ever ulcer or with a recurrent ulcer?]
> [Pause, listen, probe: is there any difference between these two types of patients regarding their understanding about the care they should be receiving or about correct self-management of the condition? In essence, does someone need to get an ulcer to learn what they need about diabetic foot ulcers?]

Do you have access to the latest NICE guidelines for the diagnosis and treatment of diabetic foot ulcers?

Do you use the latest NICE guidelines as part of your practice?

[Pause, listen, if **YES** probe: How often do you use/consult the NICE guidelines?]

[Pause, listen, probe: Did you have to look for these guidelines yourself or were they provided to you from your trust?]

[Pause, listen, probe: If there is an update or a change would you learn about it easily and quickly?]

[Pause, listen, if **NO** probe: What prevents you from using them?

[Pause, listen, probe: If there is an update or a change do you think that would you learn about it easily and quickly?]

[Pause, listen, probe: why do you say that?]

## 2. Section 2 data

Do you have all the necessary patient data readily available to you to make informed decisions about the care plan of your diabetic foot ulcer patients?

[Pause, listen, if yes probe: is it easy to access this data or do you need to login to 5 different systems?]

[Pause, listen, if no probe: What is the most important problem?]

Are there any other problems regarding access to patient data?

[Pause, listen, probe: how? Why? Is there something missing?]

Do you feel that patient data and information is efficiently shared between services and healthcare professionals?

[Pause, listen, probe.]

Is it likely that one healthcare professional might end up contradict another because of lack of effective communication?

[Pause, listen, probe.]

Is there something that could have been better regarding access to patient data and communication between professions, settings (primary, community and acute) and services?

[Pause, listen, probe.]

## 3. Section 3 referrals

Have you ever referred a patient to another service for a treatment or assessment relevant to diabetic foot ulceration?

[Pause, listen, if yes probe: Are the referrals in general quick and easy to do?]

[Pause, listen, probe: If any, what is the most important problem you have with referrals?]

Do you normally have correspondence with the service you referred the patient to in advance?

[Pause, listen, if yes probe: How does this look like?]

[Pause, listen, if no probe: Why not?]

Do you normally see the patient you referred to again?
[Pause, listen, if yes probe: How does this look like?]
[Pause, listen, if no probe: Why not?]

Do your patients ever ask you to refer them to a specific service for specialised care for diabetic foot complications?
[Pause, listen, probe. Do you think it is also part of their responsibility as patients to ask for it?]

Based on your experience do people who need specialised care access it when they need it?
[Pause, listen, if **NOT** probe. Why not?]

## 4. Section 4 ED&I

Are you aware of the notion of unconscious bias?
[Pause, listen, if yes probe: What does it mean for you?]

Do you care for people from different socioeconomic and cultural backgrounds?
[Pause, listen, probe: What type of backgrounds? Why do you say so?]

Do you believe NHS treats people from different socioeconomic and cultural backgrounds equally?
[Pause, listen, probe.]
Do you believe that the NHS treats them effectively to the same degree?
[Pause, listen, probe: What can the NHS do better?]

Are you aware of any statistics for diabetic foot ulcers based on patient demographic characteristics?
[Pause, listen, probe.]

From your experience, have you personally noticed any difference regarding the outcome of diabetic foot ulceration based on their demographic characteristics?
[Pause, listen, probe: do you think that this is what the patient believe?]
*Note for interviewer: [this = depends on what they said either YES there is discrimination or NO there is no discrimination or MAYBE there is discrimination]*

Has any patient ever spoke to you about being treated differently or being let down because of their demographic characteristics?
[Pause, listen, if yes probe: What did you do?]
[Pause, listen, probe: did you believe them?]
[Pause, listen, if no probe: Why not?]

Do you think patients treat you differently based on whether you have similar demographic characteristics as them?
[Pause, listen, probe.]

In your opinion, how can we make more people aware of any Equality Diversity & Inclusion issues and how can we improve them?
[Pause, listen, probe.]

Are you involved in patient education as part of your work?
[Pause, listen, probe.]

In your opinion, is patient education regarding diabetic foot ulceration given at the right time and in the right way to be effective?
[Pause, listen, probe.]

Is the role of carers or of family members understood and utilised appropriately (especially in people with diverse ethnic and cultural backgrounds)? For example, when you see a patient coming to see you with members of their family (mother with older children, wife with husband etc.) is this a good or the bad thing?
[Pause, listen, probe.]

## 5. Section 5: Research

Have you ever been involved in clinical research?
[Pause, listen, probe. If YES Was it about diabetes or diabetic foot?]

Do you think it is worth for you personally to be involved in research?
[Pause, listen, probe: Why?]

If they think it is worth but haven't been, then why do they think this is the case?
[Pause, listen, probe.]

How would you like to be involved?
[Pause, listen, if yes probe: Do you have a specific role in mind?]
[Pause, listen, if no probe: Are there any barriers stopping you?]

In your opinion what should we do to involve more frontline clinicians into research?
[Pause, listen, probe.]

What would entice/motivate you personally to take part in research?
[Pause, listen, probe.]

Do you think that it is hard to recruit patients for clinical research?
[Pause, listen, probe.]

In your opinion what can we do to recruit more patients in diabetes research?
[Pause, listen, probe.]

In your opinion what would entice/motivate your patients to take part in research?
[Pause, listen, probe.]

## 6. Close

Would you like to make any other comments about your experiences treating diabetic ulcers and research recruitment and participation?

Would you be willing to participate in any follow-up research?
[If so, confirm contact details.]

Thank you again for taking the time to do this interview. As a reminder, all interviews will be analysed anonymously and we will not identify you in any published report.
